# Prediction of blood pressure change during surgical incision under opioid analgesia using sympathetic response evoking threshold

**DOI:** 10.1038/s41598-021-87636-7

**Published:** 2021-05-05

**Authors:** Satoshi Kamiya, Ryuji Nakamura, Noboru Saeki, Takashi Kondo, Hirotsugu Miyoshi, Soushi Narasaki, Atsushi Morio, Masashi Kawamoto, Harutoyo Hirano, Toshio Tsuji, Yasuo M. Tsutsumi

**Affiliations:** 1 Department of Anesthesiology and Critical Care, Hiroshima University, 1-2-3 Kasumi, Minami, Hiroshima, 734-8551 Japan; 2 Medical Corporation JR Hiroshima Hospital, Hiroshima, Japan; 3 Academic Institute, College of Engineering, Shizuoka University, Hamamatsu, Japan; 4 Graduate School of Advanced Science and Engineering, Hiroshima University, Hiroshima, Japan

**Keywords:** Therapeutics, Medical research

## Abstract

Opioid inhibition of nociceptive stimuli varies in individuals and is difficult to titrate. We have reported the vascular stiffness value (K) as a standard monitor to quantify sympathetic response with high accuracy. On the contrary, among individuals, a considerable variation in the rate of change in K for constant pain has been observed. In this study, we proposed a new index, the minimum stimulus intensity value that evoked the response on K (MEC_K_: Minimum Evoked Current of K), and evaluated its accuracy in predicting sympathetic response to nociceptive stimuli under constant opioid administration. Thirty patients undergoing open surgery under general anesthesia were included. After anesthetic induction, remifentanil was administered at a constant concentration of 2 ng/ml at the effect site followed by tetanus stimulation. MEC_K_ was defined as the minimal current needed to produce a change in K. MEC_K_ significantly (*P* < 0.001) correlated with the rate of change of systolic blood pressure during skin incision (ROC_BP_). Bland–Altman plot analysis using the predicted ROC_BP_ calculated from MEC_K_ and the measured ROC_BP_ showed that the prediction equation for ROC_BP_ was highly accurate. This study showed the potential of MEC_K_ to predict blood pressure change during surgical incision under opioid analgesia.

Clinical trial registration Registry: University hospital medical information network; Registration number: UMIN000041816; Principal investigator's name: Satoshi Kamiya; Date of registration: July 9th, 2019.

## Introduction

The International Association for the Study of Pain defines pain as an aversive sensory and emotional experience. Patients under general anesthesia are unconscious and do not have an aversive experience. However, anesthesiologists commonly use analgesics in addition to sedatives because nociceptive stimuli can cause noxious autonomic reflexes. Opioids are the most commonly used analgesics during general anesthesia due to their lack of a ceiling effect. Opioids exert their analgesic effects mainly by inhibiting sensory nerve transmission in the spinal cord and by inhibiting the excitation of pain conduction pathways in the brain. When opioids are administered during general anesthesia, noxious autonomic reflexes are suppressed and multiple parameters such as heart rate, blood pressure, electrocardiogram, and respiratory rate are affected. During surgery, anesthesiologists rely on these parameters to estimate pain levels and adjust the opioid dosage. However, this nociceptive stimuli-induced sympathetic response varies among individuals, and therefore, so do individual anesthetic requirements. Adverse events, either as an unexpected increase in blood pressure due to underdosing or delayed arousal due to overdosing, are common. Thus, if the individual's opioid requirement can be accurately quantified in advance, more stable general anesthesia can be performed.

Nociceptive stimuli input to the central nervous system is output to effector organs such as the heart and blood vessels via the sympathetic nervous system. Opioids inhibit the input of nociceptive stimuli to the central nervous system. Therefore, the administration of opioids blunts the sympathetic response to nociceptive stimuli. In other words, by accurately quantifying the sympathetic response to a given nociceptive stimulus under opioid administration, we can determine the relationship between the opioid dose and the response to the nociceptive stimulus and quantify opioid sensitivity. Photoplethysmography (PPG), an increasingly popular tool, has recently begun to be used to measure sympathetic cutaneous blood flow responses for quantifying sympathetic nerve activity in peripheral vasculature^1–4^. However, PPG measures blood flow at the measurement site and does not, in principle, directly indicate the degree of sympathetic response. Therefore, we proposed a method to extract only the degree of vasoconstriction from sympathetic cutaneous blood flow responses to PPG and quantify it as vascular stiffness value (K)^5^. We reported that K could be used to quantify pain and reflect the activity of the sympathetic nervous system^6,7^. Moreover, we reported that K value reflects changes in pain caused by the administration of opioids during general anesthesia and that the response of K to pain was attenuated with increasing doses of opioids^8–10^.

On the contrary, among individuals, a considerable variation in the rate of change in K for constant pain has been observed^9,10^. We speculated that this may have been due to the fact that the pathway of nociceptive stimuli, which travel from the peripheral nerves through the central nervous system to effector organs such as peripheral blood vessels, is strongly influenced by autonomic changes due to aging and coexisting diseases. While opioids inhibit the afferent pathway of pain perception, the rate of change of K represents the intensity of the sympathetic response or the efferent pathway. In other words, the rate of change in K includes information from both efferent and afferent pathways, so the measurement results may vary depending on the sensitivity of the effector. Therefore, we hypothesized that the “intensity of nociceptive stimuli" at which sympathetic responses appear is a better indicator of opioid sensitivity than the "intensity of sympathetic responses" to nociceptive stimuli (that is, the rate of change in K) because it exclusively extracts only information regarding afferent pathways.

In this study, we proposed a new index, the minimum stimulus intensity value that evoked the response (MEC: Minimum Evoked Current ) on each parameter, and evaluated its accuracy in predicting sympathetic response to nociceptive stimuli under constant opioid administration. The primary objective of this study was to compare the prediction accuracy of ROC_BP_ by MEC for each parameter, and the secondary objective was to compare the prediction accuracy of ROC_BP_ between MEC_K_ and K_R80_.

## Patients and methods

### Patients

Prior to the study, we received approval from the Ethics Committee of Hiroshima University (‘Hi’-226, ‘E’-1523-1) and registered the clinical trial (registry: university hospital medical information network, registration number: UMIN000041816, principal investigator's name: Satoshi Kamiya, Date of registration: July 9th, 2019). This study was conducted in accordance with the Declaration of Helsinki and STROBE statement. The study population consisted of patients over 20 years of age who underwent open surgery under general anesthesia from July 2019 to October 2019. A total of 30 patients gave their written informed consent before the study. All procedures were conducted at the Hiroshima University Hospital. We excluded patients with irregular R-R via electrocardiogram (ECG), inability to perform invasive arterial pressure measurements in the radial artery, significant hemodynamic or neurological impairment in the upper extremity, and severe stenosis or occlusive lesions in the coronary arteries or cerebral vessels. If, for a patient, the mean blood pressure remained below 50 mmHg for more than 3 min during the study time, the study protocol for that patient was discontinued.

### Measurement and protocol

Before induction of anesthesia, a photoplethysmography probe (TL-271T, NIHON KODEN, Tokyo, Japan) on the middle finger of the left hand, ECG on the chest, electroencephalogram (EEG; Entropy, GE HEALTHCARE UK LTD., Buckinghamshire, UK) on the anterior forehead, neuromuscular blockade monitoring device (NMT- Neuromuscular Transmission, GE HEALTHCARE UK LTD., Buckinghamshire, UK) on the ulnar side of the forearm of the right hand, and a non-invasive blood pressure cuff on the right upper arm were placed. In all patients, a preoperative dosing plan was developed to achieve a predicted effect-site concentration of 2 ng/ml remifentanil. Minto's pharmacokinetic model^11^ was used to calculate predicted effect-site concentrations. Administration of remifentanil was initiated as per the dosing plan, and propofol 3 μg/ml was used to induce anesthesia using a target-controlled infusion (TCI) pump with built-in ‘Diprifusor’ (TE-371, TERUMO, Tokyo, Japan). After the patient was unconscious, 50 mg of rocuronium was administered and a 22 G catheter was secured in the left radial artery for measuring arterial blood pressure (ABP). Data from ECG, ABP, and PPG were output to a personal computer from a bedside patient monitor (BSS-9800, NIHON KODEN, Tokyo, Japan) and were used to calculate K values in real-time.

After the predicted effect-site concentration of remifentanil reached a steady state at 2 ng/ml, tetanus stimuli at 50 Hz for 5 s were delivered through a two-pole body surface electrode on the ulnar side of the right hand using the INNERVATOR 252 (FISHER & PAYKEL HEALTHCARE, Auckland, New Zealand). The current value was initially 10 mA and increased in increments of 10 mA until 80 mA, the maximum output of the INNERVATOR 252, was reached. Thus, a total of eight tetanus stimulation sessions were performed. Thirty seconds after the tetanus stimulation, and after at least another 10 s of steady-state, the next stimulation was performed. Subsequently, tracheal intubation was performed using a Macintosh laryngoscope or McGRATH MAC video laryngoscope (MEDTRONIC, Dublin, Ireland). All endotracheal intubations were successfully executed in a single attempt. Thereafter, the same amount of propofol TCI target blood levels and rate of remifentanil administration were maintained until the skin incision was performed.

### Calculation of K

**Figure 1:**
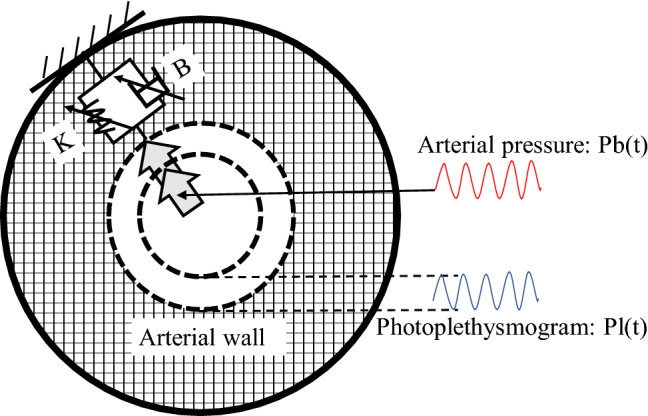
Schematic diagram of the mechanical impedance model. The model used in this study consists of a parallel arrangement of springs and dampers across the diameter of the arterial wall. When a force (blood pressure) is applied across the diameter of the artery, the arterial wall is dampened by springs and dampers that represent the rigidity and viscosity of the arterial wall itself. The behavior of the arterial wall after damping is observed as a change in the photoplethysmogram waveform.

In this study, the motion of the vessel wall was predicted using a model in which springs and dampers were aligned in parallel to the diameter direction of the vessel. The arterial wall, moved by a force (blood pressure) applied in the direction of the diameter of the vessel, was dampened by springs and dampers that represent the stiffness and viscosity of the vessel wall itself. The behavior of the vessel wall after damping was observed as a change in the PPG waveform (Fig. 1). We used the spring constant in this model equal to K. Details of the calculation of K value have been reported previously^8^. In brief, we first detected the heartbeats with the R wave of the ECG and cut out the ABP and PPG waveforms for each heartbeat. All wave height data associated with one heartbeat were provided in the following equations:

1 $$ {{\rm dPb}}\left( t \right) = {{\rm KdPl}}\left( t \right) + {{\rm Bd}}\dot{\mathrm{P}}{{\rm l}}\left( t \right), $$

2 $$ {{\rm dPb}}\left( t \right) = {{\rm Pb}}\left( t \right) - {{\rm Pb}}\left( {t_{0} } \right),\;{{\rm  dPl}}\left( t \right) = {{\rm Pl}}\left( t \right) - {{\rm Pl}}\left( {t_{0} } \right), $$

3 $$ {{\rm d}}\dot{{\rm P}}{{\rm l}}\left( t \right) = {\dot{{\rm P}}{\rm l}}\left( t \right) - {\dot{{\rm P}}{{\rm l}}}\left( {t_{0} } \right), $$

The time at which the change was first observed was considered *t*_0_, ABP wave height at time *t* was Pb(*t*), and PPG wave height at time *t* was represented as Pl(*t*). Ṗl(*t*) is equal to the first derivative of the PPG wave height. The coefficients for vascular stiffness and vascular viscosity values, K and B, respectively, were determined as one value per heartbeat by performing a least-squares fit of the equation to all data acquired in each heartbeat. K determined in this way was actually displayed automatically at the bedside in real-time, every heartbeat. If the coefficient of determination was less than 0.95, or if K and/or B were negative, the data were removed from the analysis. Only K was analyzed in the present study because previous studies have estimated that K was a promising candidate as a measure of nociceptive stimulus intensity^8^.

### Statistical analysis

**Figure 2:**
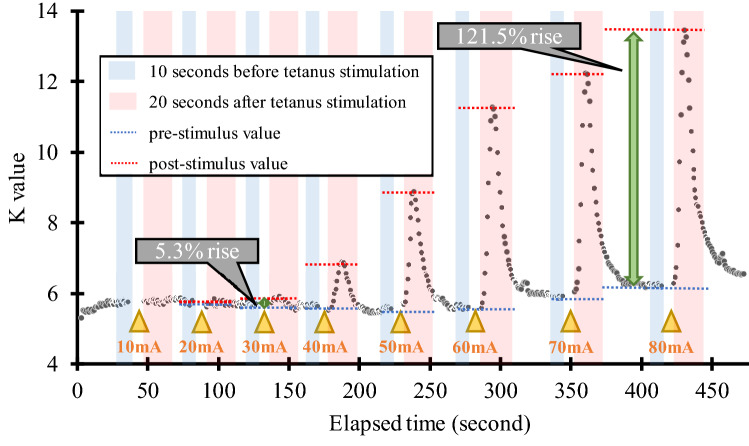
Schema describing minimum evoked current (MEC) measurement in the study. An example of variation in vascular stiffness value (K) during tetanus stimulation. The predicted effect-site concentration of remifentanil was maintained at 2 ng/ml throughout the study. In the figure, △ indicates the timing of tetanus stimulation, which was incrementally increased by 10 mA. The rate of K change increased with increasing stimulus intensity. In this case, the first time the rate change of K exceeded 5% was after 30 mA stimulation (MEC_K_ = 30 mA). The rate of change in K at 80 mA is 121.5%, which is K_R80_.

Figure 2 shows an example of how the MEC was measured in this study. Each parameter measurement in tetanus stimulation is described in the following text. First, pre-stimulus values were set as the median value determined throughout the 10 s that preceded tetanus stimulation. Post-stimulus values were the maximum values of K, heart rate, systolic blood pressure, and minimum values of PPG amplitude for 20 s after tetanus stimulation. The post-stimulus values were divided by the pre-stimulus values and the percentage change in parameters in response to tetanus stimulation at each stimulus intensity was calculated. MEC was defined as the minimum stimulus intensity value that produced a change of more than 5% in the parameter during tetanus stimulation. However, once the change was greater than 5%, and if the rate of change was less than 5% at a subsequent stage, the MEC calculated at a lower current value was rejected as noise. In other words, the MEC would eventually be one step stronger than the maximum stimulus intensity at each stimulus intensity where the rate of change of the parameter was below 5%. If no more than 5% change appeared at 80 mA tetanus stimulation, MEC was classified as outside the measurement range. The MEC for K, HR, BP, and PPG were designated as MEC_K_, MEC_HR_, MEC_BP_, and MEC_PPG_, respectively. The rate of change in K before and after 80 mA tetanus stimulation (K_R80_) was calculated as a measure of the rate of change for constant intensity nociceptive stimuli. The median systolic blood pressure of 10 s before the scalpel skin incision was defined as pre-BP, and the maximum systolic blood pressure between the skin incision and the start of electrocautery use was defined as post-BP. The ROC_BP_ before and after skin incision was calculated by dividing post-BP by pre-BP. A first-order regression was performed to confirm the relationship between MEC and ROC_BP_. Specifically, a first-order regression equation was created between cases where MEC and ROC_BP_ could be measured. A Smirnov–Grubbs test was performed using this regression formula, and a measurement in which P was greater than or equal to 0.05 was removed as an outlier. The regression equation was remade for each of the removed outliers. The regression equation at the time outliers were removed was adopted as the final correlation line. Pearson's correlation coefficients were calculated using the measurements at the time when the outliers were eliminated. Adjusting for multiplicity, p values of Pearson's correlation coefficients were corrected using the Bonferroni correction 4 times. The significance level was set at 0.05.

Further, we attempted to predict the ROC_BP_ using MEC_K_ and K_R80_. Specifically, the prediction equation was based on the first-order approximate equation previously developed, and the predicted ROC_BP_ was calculated from MEC_K_. For cases in which MEC_K_ was outside the measured range, we fitted the mean value of ROC_BP_ in out-of-range cases to the approximate equation and substituted the estimated value. A similar first-order approximation and outlier treatment was performed for K_R80_ and ROC_BP_. From the final approximate equation, the predicted ROC_BP_ was calculated. A Bland–Altman analysis was performed for both measured and predicted ROC_BP_, and fixed bias and limit of agreement range were calculated for each.

No prior studies are available on MEC_K_, and therefore, the prior number of cases cannot be determined. Power analysis showed that 26 patients were needed to show Pearson’s correlation coefficient of 0.5 between the K and BP under the conditions α = 0.05 and 1-β = 0.8. The number of patients was set to 30 to account for the fact that some data measurements were incomplete. Moreover, the effect size was calculated to ensure a sufficient sample size. In Pearson's correlation analysis, the r-value, which is the correlation coefficient, was used as a measure of the effect size. All the r-values of the main results of this study indicate moderate-to-high effect sizes.

## Results

A total of 30 patients (male, 15) were included in the study and the mean age of participants was 62 years. No patients who regularly used opioids were included. Thirteen patients underwent an upper abdominal incision, and 17 underwent a lower abdominal incision. None of the patients met the exclusion criteria. A summary of patient characteristics is shown in Table 1. ROC_BP_ was 18.3 ± 11.8% (mean ± SD).

**Table 1:** Patient characteristics.

Male/Female	15/15
Age (years)	62 ± 13(35–80)
Height (cm)	161.7 ± 9.7(143.3–177.8)
Weight (kg)	56.3 ± 13.3(36.9–99.6)
BMI (kg/m^2^)	21.6 ± 4.8(12.5–39.9)
Upper/lower abdomen	13/17
sBP pre-incision(mmHg)	77.8 ± 13.3(56.0–108.0)
sBP post-incision(mmHg)	91.7 ± 16.1(57.4–126.0)
Rate of change in sBP before and after incision (%)	18.3 ± 11.8 (− 3.7–38.4)

Results are presented as mean ± SD (minimum–maximum).

*BMI* body mass index, *sBP* systolic blood pressure.

**Figure 3:**
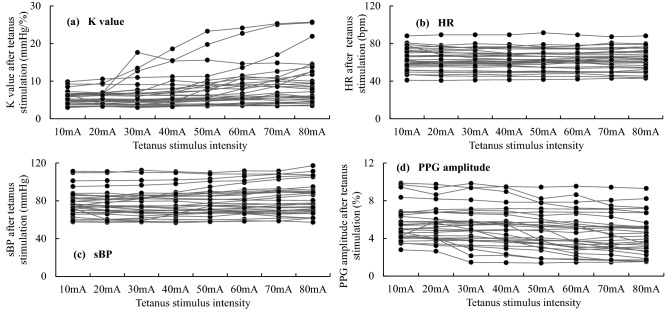
vascular stiffness value (K), systolic blood pressure (sBP), heart rate (HR), and photoplethysmography amplitude (PPG) after tetanus stimulation at each intensity. There were many cases in which the value of K increased with increasing stimulus intensity. However, when the stimulus intensity was large, individual differences in the K change tended to be large. Values of PPG amplitude tended to decrease with increasing stimulus intensity. In many cases, there was no obvious change in HR even when the stimulus intensity was increased.

**Figure 4:**
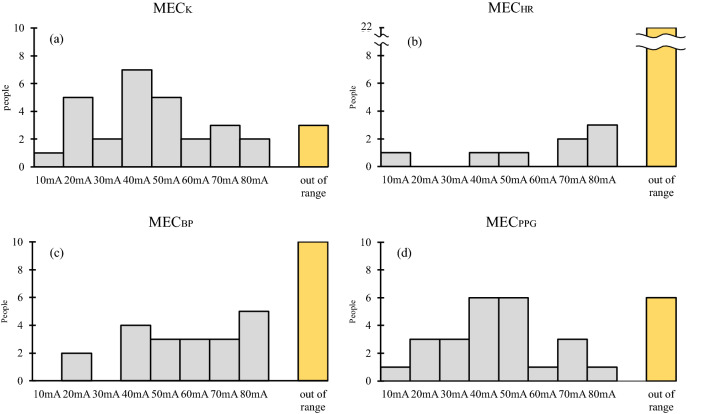
Histogram of each minimum evoked current (MEC). The distributions of MEC, vascular stiffness value (K), systolic blood pressure (BP), heart rate (HR), and photoplethysmography amplitude (PPG) are shown. MEC represents the minimum stimulus current intensity at which a change of more than 5% in the parameter appears for tetanus stimuli given in 10 mA increments. Except for HR, which was very often outside the measurement range, frequency peaks were generally around 40–50 mA.

Figure 3 shows the values of K, HR, sBP, and PPG amplitude after tetanus stimulation of each intensity. K and sBP values tended to increase with the intensity of the stimulus current. PPG amplitude tended to decrease with increasing stimulus intensity. HR did not show consistent changes. Even for K, which produced the least individual differences, actual measured values had a large degree of individual variability. Therefore, it was not possible to detect the presence of a change in K by setting a threshold for the actual measured value of K. Next, MEC was determined based on the rate of change between each measured value and its pre-stimulus value. Figure 4 shows a histogram of the four MEC. MEC could be measured in 27 cases for K, 8 for HR, 21 for BP, and 25 for PPG. The frequency peaks were generally in the vicinity of 40–50 mA, except for HR, where MEC was very often outside the measurement range.

**Figure 5:**
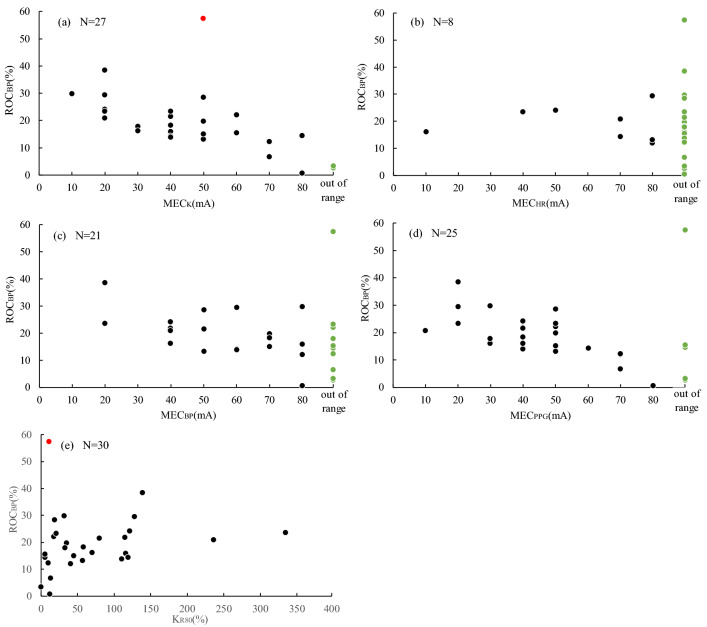
Relationship between minimum evoked current (MEC) + rate of change in K for 80 mA tetanus stimulation (K_R80_) and rate of change of systolic blood pressure (ROC_BP_). A scatter plot of each minimum evoked current (MEC), vascular stiffness value (K), systolic blood pressure (sBP), heart rate (HR), photoplethysmography amplitude (PPG), and rate of change of systolic blood pressure (ROC_BP_) is shown. Out-of-range cases are shown using green points, and Smirnoff-Grubbs test outlier points are shown in red. A downward rightward trend is observed for MEC_K_, MEC_BP_, and MEC_PPG_, and an upward rightward trend is observed for K_R80_.

Then, in addition to the MEC of each parameter determined above, K_R80_, the rate of change in K at the maximum current value (80 mA), was calculated. A scatter plot of each MEC and K_R80_ with ROC_BP_, respectively, is depicted in Fig. 5. Pearson's correlation for each MEC and ROC_BP_ is − 0.723 (MEC_K_), − 0.067 (MEC_HR_), − 0.565 (MEC_BP_), − 0.711 (MEC_PPG_) (*P* < 0.001, *P* = 0.87, *P* = 0.009, and *P* < 0.001, respectively). MEC_K_, MEC_BP_, and MEC_PPG_ were shown to be significantly correlated with ROC_BP_. Three cases for MEC_K_, 9 cases for MEC_BP,_ and 5 cases for MEC_PPG_ were classified as out of the measurement range. The ROC_BP_ of these out-of-range cases were 2.6% (− 3.7 to 3.3), 14.4% (2.6–23.3), and 3.3% (− 3.7 to 15.4), respectively.

Among the out-of-measurement range groups, there was no statistically significant difference in the ROC_BP_ of MEC_K_ and MEC_PPG_ measurement range groups. However, out-of-range cases of MEC_PPG_ had a large degree of variability and included cases with large ROC_BP_. Therefore, we determined that MEC_K_ was the index with the highest degree of correlation with ROC_BP_ among MEC_K_, MEC_HR_, MEC_BP_, and MEC_PPG_.

We then used MEC_K_ and K_R80_ to predict ROC_BP_. First, for MEC_K_, a first-order regression equation was applied after outlier treatment in the scatter plot in Fig. 5a, as follows:

4 $$ {{\rm Predicted}}\,{{\rm ROC}}_{{{{\rm BP}}}} \,\left( \% \right) = - 0.28 \times {{\rm  MEC}}_{{{\rm K}}} \,\left( {{{\rm mA}}} \right) + {31}.{26}\;\left( {{{\rm r}} = 0.{723},\;P < 0.00{1}} \right), $$

where the mean value of ROC_BP_ in cases outside the MEC_K_ measurement range was 0.7%. This rate of change was substituted into the temporal regression equation, which yielded a value of 107 (mA). This value was used as a provisional MEC_K_ value for cases outside the MEC_K_ measurement range. Next, for K_R80_, the first-order regression equation after outlier treatment in the scatter plot in Fig. 5e was applied, as follows:

5 $${{\rm Predicted}}\,{{\rm ROC}}_{{{{\rm BP}}}} \,(\% )= 0.05 \times {{\rm K}}_{{{{\rm R8}}0}} \,\left( \% \right) + 13.25\;\left( {{{\rm r}} = 0.{441},\;P = 0.017} \right). $$

**Figure 6:**
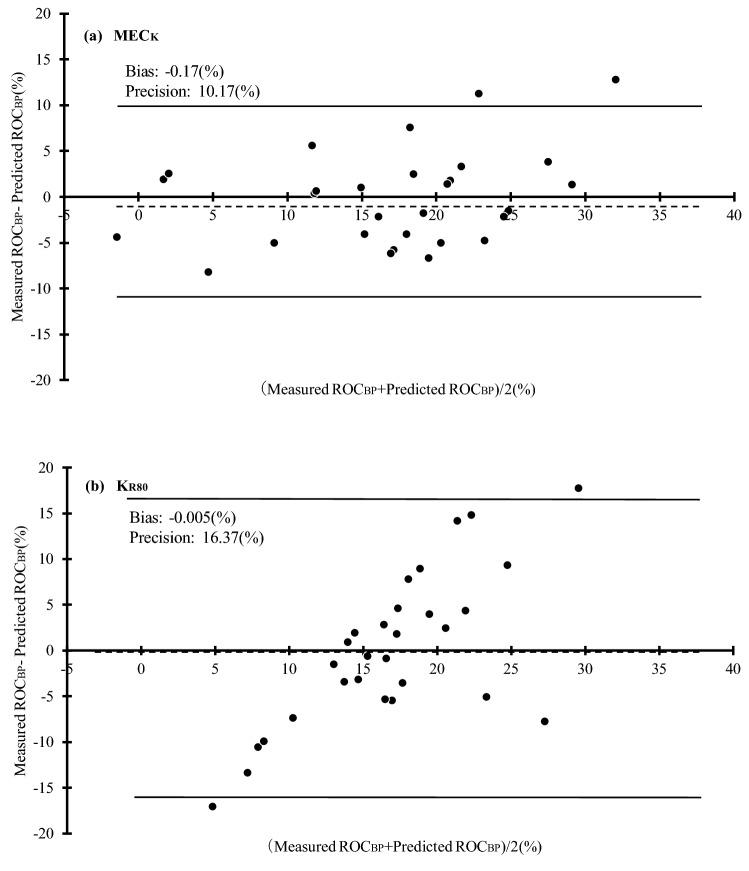
Comparison of the accuracy of ROC_BP_ predictions using MEC_K_ (**a**) and K_R80_ (**b**). The error associated with predicted and measured rates of change of systolic blood pressure (ROC_BP_) estimated by MEC_K_ and K_R80_, respectively, are shown. The fixed bias was determined to be small since the data used to create the prediction equation, and data used for validation were the same. The proportional error of MEC_K_ was small. K_R80_ displayed a definite proportional error, and its prediction performance was determined to be low.

The above prediction equation was used to calculate the predicted ROC_BP_. The results of the Bland–Altman plot of measured and predicted values are shown in Fig. 6. The difference between measured and predicted ROC_BP_ is shown as the vertical axis and the mean as the horizontal axis. For MEC_K_, fixed bias was small (− 0.17%), and the Pearson correlation coefficient in the scatter plot was R = 0.322 (*P* = 0.088), and thus, no significant proportional bias was found. In contrast, for K_R80_, the fixed bias was small (− 0.01%); however, the Pearson correlation coefficient of the scatter plot contained significant proportional bias (R = 0.714, *P* < 0.001).

## Discussion

Although opioids are essential drugs for general anesthesia, inappropriate administration may lead to unpleasant effects on patients. Estimation of appropriate dosage is difficult because of large individual differences in opioid sensitivity. In this study, a prediction formula using MEC_K_ as an indicator was able to predict ROC_BP_ with high accuracy.

MEC_K_ is the threshold for the appearance of a sympathetic response when blood concentrations of opioids are 2 ng/ml and can be regarded as an intrinsic value that indicates individual opioid sensitivity. In other words, for patients with a low MEC_K_, increasing the dose of opioids prior to skin incision can reduce circulatory fluctuations. The relationship between the MEC_K_ and ROC_BP_ at different opioid concentrations remains to be determined; however, the present results indicate that the MEC_K_ may be a good indicator of opioid sensitivity.

### MEC_K_ and K_R80_

MEC_K_ correlated better with ROC_BP_ than K_R80_ (Fig. 5) and precision was also small (Fig. 6). This implies that MEC_K_ was more accurate when predicting ROC_BP_ than K_R80_. We previously reported that the rate of change in K with constant pain was inversely related to opioid concentration. However, there were large individual differences in the relationship between opioid concentration and the rate of change in K^9^. This may be due to individual differences in sympathetic response to noxious stimuli. When the primary afferent sensory nerve was depolarized by nociceptive stimulation, the signal was transmitted to the hypothalamus by the depolarization of the secondary afferent sensory nerve. A nociceptive signal input to the hypothalamus was transmitted to the sympathetic nerve center, and an efferent signal caused an autonomic nervous reaction such as blood vessel contraction, blood pressure increase, and heart rate change. Opioids suppress autonomic nervous responses by various actions such as transmitter release of primary afferent sensory nerves, suppression of depolarization of secondary afferent sensory nerves, and activation of the descending pain inhibitory system by disinhibition of the hypothalamus. The index of the response strength of K itself, such as K_R80_, measures the strength of the response when the noxious stimulus is transmitted to the sympathetic nerve center via the afferent and further to the peripheral effector through the centrifugal tract. Naturally, the presence or absence of afferent stimuli alters K_R80_; however, at the same time differences in the responsiveness of the effector also alter K_R80_. In contrast, MEC_K_ is independent of effector responsiveness because it measures the presence or absence of sympathetic responses to nociceptive stimuli. Therefore, MEC_K_ could predict blood pressure fluctuations associated with skin incision more accurately than the K_R80_.

### Correlation with ROC_BP_ during skin incision

MEC_K_ and MEC_PPG_ correlated better with ROC_BP_ versus MEC_HR_ and MEC_BP_, which suggests that MEC_K_ and MEC_PPG_ may capture sympathetic responses to nociceptive stimuli more accurately. In contrast, the number of cases outside the measurement range of MEC_PPG_ was higher than that of the MEC_K_ (6 vs 3 for MEC_PPG_ and MEC_K_, respectively), and ROC_BP_ variability of MEC_PPG_ was also higher. This may be because PPG amplitude reflects changes in blood flow and is influenced by multiple parameters such as cardiac output and vascular tone. The PPG amplitude was reduced, probably by vasoconstriction with nociceptive stimuli; however, it may have been increased despite nociceptive stimuli as a consequence of an augmentation factor that was a result of increased cardiac output that occurred at the same time^12^.

In contrast, K was not affected by blood flow changes due to changes in cardiac output because it measured vascular compliance. This means that in cases where PPG cannot detect or shows excessive changes, K can accurately detect the presence or absence of a sympathetic response. For these reasons, we believe that the MEC_K_ was the most accurate measure of MEC.

### Stimulation algorithm

In this study, we used a tetanus stimulation of 50 Hz for 5 s. This is shorter than a stimulation interval of 30 s reported in a previous study^13^. It is possible that the short stimulation time increased the number of out-of-range groups, especially in MEC_HR_. However, when nociceptive stimulation is given under opioid analgesia, the possibility may arise that sympathetic responses do not occur in patients with high opioid sensitivity. Considering that more than 80% of BP and more than 60% of PPG measurements could measure MEC by under 80 mA tetanus stimulation and that a significant correlation was observed between MEC_BP_, MEC_PPG_ and ROC_BP_, we regarded this as an appropriate tetanus stimulation time for BP and PPG.

Sympathetic response also occurs in muscle contraction induced by tetanus stimulation. Therefore, tetanus stimulation is not considered to be a pure Aδ or C fiber stimulus. However, Funcke et al. stated that the sympathetic responses to tetanus and intradermal electrical stimulation might be considered as a similar stimulus^13^. In this study, the tetanus stimulus was highly specific to Aδ and C fibers in the inhibition of muscle contraction due to the administration of muscle relaxants. However, it is not ruled out that repeated tetanus stimulation may cause neural sensitization, which may modulate the measurement results at the next stimulation. K_R80_, which indicates the intensity of the sympathetic response, may be affected by neurosensitization. In contrast, it is unlikely that MEC was affected by neurosensitization because stimuli weaker than MEC did not affect each parameter.

### Limitations

To generalize the opioid sensitivity index based on MEC_K_, additional studies designed to adjust the remifentanil dose using MEC_K_ as an indicator are needed. In addition, since the results of this study strictly apply only to propofol anesthesia, it is also necessary to verify whether the results are applicable when anesthetics other than propofol are used or when anesthesia is not performed.

Since the remifentanil concentration was studied only at 2 ng/ml, the changes in MEC and ROC_BP_ at other concentrations are unknown. However, at 2 ng/ml, MEC varied appropriately between 10 and 80 mA. In a previous study by our research group, 6 ng/ml resulted in many cases with very poor sympathetic responses^9^. In contrast, using a remifentanil concentration lower than 2 ng/ml may result in too high ROC_BP_, which seems not to be appropriate for patient safety. These facts suggest that the concentration of 2 ng/ml used in this study was reasonable regarding the amount of opioids needed to inhibit tetanus stimulation.

In the present study, the interval between stimuli (30 s) was shorter than that reported by Funke et al.^13^, and the median value of 10 s before stimulation was set as the pre-stimulus value. Therefore, it is possible that return to the steady-state was not reliably judged. However, prolonging the study time under general anesthesia increases the possibility of external factors such as changes in body temperature and insensible perspiration, etc. Since measurements including K can be taken in real-time for each heartbeat, we consider that the effect of the short interval between stimuli may be more limited than that reported in the past literature.

In the present study, the peak value for 20 s after the stimulus was adopted as the post-stimulus value. This is shorter than the value reported in previous studies. The peak values of blood pressure and heart rate caused by the increase in blood catecholamine concentration due to nociception may occur later than 20 s. However, the time from tetanus stimulation to the peak value in the cases where we were able to set MEC was 9.8 ± 1.9 s, 7.3 ± 1.4 s, 10.0 ± 2.0 s, and 9.9 ± 1.9 s (mean ± standard deviation) for K, HR, sBP, and PPG, respectively. In other words, the neurogenic response peaked within 20 s for all parameters. Therefore, we believe that ending the measurement at 20 sseconds has a limited impact on MEC, which is the essence of this study.

Since the measurement of arterial pressure requires an invasive blood pressure measurement method and takes time, it is generally difficult to accept this research model as-is for clinical use. In the future, it will be necessary to improve the protocol by reducing the number of stimulation steps at low currents, where responses are less likely to occur.

## Conclusions

Compared with K_R80_, MEC_HR_, MEC_BP_, and MEC_PPG_, MEC_K_ correlated best with blood pressure changes during skin incisions. The MEC_K_-indexed prediction equation adequately predicted the increase in blood pressure that occurred during skin incision at a remifentanil effect-site concentration of 2 ng/ml. MEC_K_ was shown to be a potentially more accurate indicator of opioid sensitivity than the rate of change in K.
